# Nanostructured and Photochromic Material for Environmental Detection of Metal Ions

**DOI:** 10.3390/molecules24234243

**Published:** 2019-11-21

**Authors:** Raphael C. L. Machado, Frank Alexis, Frederico B. De Sousa

**Affiliations:** 1 Laboratório de Sistemas Poliméricos e Supramoleculares, Instituto de Física e Química, Universidade Federal de Itajubá, Itajubá 37500-903, Brazil; raphaelclmachado@gmail.com; 2 School of Biological Sciences and Engineering, Yachay Tech University, San Miguel de Urcuquí, Ibarra EC 100150, Ecuador; falexis@yachaytech.edu.ec

**Keywords:** electrospinning, polymer, nanofibers, sensors, spiropyran, metal ions

## Abstract

Compared to conventional spectroscopy or chromatography analysis, chemical sensing based on colorimetric changes offers an alternative to monitor potential metal hazards in aqueous environment through rapid and low-cost colorimetric changes which can be easily interpreted. In this work poly(ethylene glycol) (PEG 2000) was modified with a carboxylic acid spiropyran (SPCOOH) derivate by Steglich esterification (PEGSP2). PEGSP2 was incorporated into a poly(ε-caprolactone) (PCL) polymer matrix by electrospinning technique to produce nanofibers with photochromic properties. Spectroscopic analysis, thermal gravimetric analysis (TGA), and differential scanning calorimetry (DSC) were used to characterize PEGSP2. Drop shape analysis (DSA) and scanning electronic microscopy (SEM) were used to characterize the electrospun (ES) nanofibers morphology. Several metal ions solutions relevant to environmental hazards were prepared to be spotted on the surface of ES nanofibers for photochromatic sensing. Among them, Mg^2+^, Ca^2+^, Zn^2+^, Cd^2+^, La^3+^, and Er^3+^ demonstrated orange fluorescence when exposed to UV light. ES nanofibers also presented higher wettability when compared to a pure PCL polymer matrix, which is critical for sensitivity. Eighteen metals ions could be detected on the electrospun material. Additionally, among all metal ions Fe^3+^ was the most sensitive one in solution, in a µmol L^−1^ range.

## 1. Introduction

Sensors provide information about the concentration of chemical, physical, and biological in certain molecules in a determined environment. Sensing of environmental hazards has been used to detect possible risks of soil, air, and water contamination. Thus, devices based on electrospun (ES) nanofibers have been made, and, chemical sensing based on polymeric nanofibers is becoming increasingly important as it offers a low-cost alternative to conventional analytical instrumentation. Chemical sensing is an area of interest for monitoring toxic gases in the air like carbon dioxide [1] or ammonia [2]. The sensing of contaminants in natural waters by industrial effluents [3] including organophosphorus, fluorinated, and pesticide compounds have also been developed to monitor potential risks [4]. There are biosensors that monitor chemical compounds that are important for human well-being such as glucose in blood samples [5] and oxygen [6]. These materials have some advantages including rapid response, reduced photochromic fatigue [7], and low cost compared to conventional analytical instruments, such as spectrophotometer or chromatograph equipment [8]. Additionally, these nanofibers can be integrated into electronic devices, and they have low energy requirements [9]. These advantages offer opportunities to use sensors in conditions that were not possible when using analytical laboratories.

Most of the sensors are polymeric devices that possess organic compounds to detect inorganic analytes or vice versa. It is widely accepted that the sensitivity of a sensor that detects analytes by interaction on the surface, will increase with increasing surface area per mass unit [10]. Nanofibers properties enhance the interaction with analytes in the gas phase such as volatile organic compounds (VOC) [11] and hydrogen chloride (HCl) [12]. There is a variety of colorimetric sensors based on nanofibers described in the literature that detect some analytes in aqueous media, including Fe^3+^ and Hg^2+^ [13], Cu^2+^ [14], Zn^2+^ [15], and Al^3+^ [16]. There is a variety of methods for the production of nanostructured materials among them wet, dry, melt, gel and force spinning [17]. These techniques allow the materials to have a high surface area to volume ratio. Electrospinning is a physical process which has been the object of growing interest due to its versatility for sub-micron fibers production Moreover, the technique combines simplicity and versatility, allowing more possibilities for the production of scalable ordered assemblies when compared to commonly used techniques [18], so electrospinning modified polymers could be a good approach for the production of nanoscaled colorimetric sensors. Our polymer functionalization consisted of a post-polymerization [19,20] esterification with a carboxylic acid spiropyran derivate to introduce the sensing group into the polymeric structure. The polymeric structure is critical for increasing the number of sensing groups and enhancing the sensitivity of the sensor.

**Figure 1:**
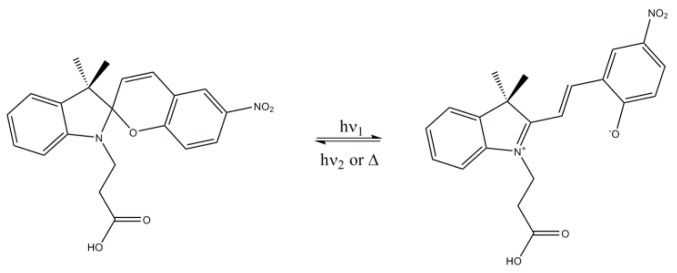
Structural transformation of an acid spiropyran derivate (SPCOOH) from SP isomer (**left**) and merocyanine (MC) isomer (**right**).

Spiropyran (SP) molecules constitute a class of organic molecules that can undergo reversible structural transformations. As a result of the isomerization phenomenon SP changes to merocyanine (MC), a colored form commonly called “open form”. Many external stimuli can trigger the phenomenon, such as radiation (ultraviolet), pH, ions, solvent polarity and even mechanical stress [21]. The structural isomerization of SP is described in the literature [22], and it is shown in Figure 1. The MC isomer is more polar than the SP form (also called “closed form”) and can exist in the quinoidal and zwitterionic forms depending on the polarity of the environment and the electronic properties of the substituent group in the chromene moiety [23]. The MC form can be converted to the SP one by visible light irradiation or heat [24].

Herein, we report a post-polymerization modification of poly(ethylene glycol) (PEG) functionalized with a (l-(β-Carboxyethyl)-3′,3′-dimethyl-6-nitrospiro(indoline-2′,2[2*H*-1] benzopyran) (SPCOOH) by Steglich [25] esterification (PEGSP2) and the preparation of photochromic ES nanofibers based on the polymer blend containing poly(ε-caprolactone) (PCL) and PEGSP2. This photochromic polymer was characterized by Fourier transform infrared spectroscopy with attenuated total reflectance (FTIR-ATR), nuclear magnetic resonance (NMR), ultraviolet-visible spectroscopy (UV-Vis), thermal gravimetric analysis (TGA/DTG) and differential scanning calorimetry (DSC). Furthermore, nanofibers were also characterized by scanning electron microscopy (SEM), drop shape analysis (DSA), FTIR-ATR and UV-vis analysis. The ES nanofibers were tested as a colorimetric sensor with several metal ions in an aqueous media, including Mg^2+^, Ca^2+^, Sr^2+^, Ba^2+^, Cr^3+^, Mn^2+^, Fe^3+^, Fe^2+^, Co^2+^, Ni^2+^, Cu^2+^, Zn^2+^, Cd^2+^, Hg^2+^, Pb^2+^, La^3+^, Ce^3+^, Nd^3+^, Eu^3+^, Er^3+^ and Yb^3+^; a colorimetric response was observed for some of them, and additionally, an orange fluorescence response under UV light was observed for Mg^2+^, Ca^2+^, Zn^2+^, Cd^2+^, La^3+^ and Er^3+^.

## 2. Results

### 2.1. PEGSP2 Structural Characterization

**Figure 2:**
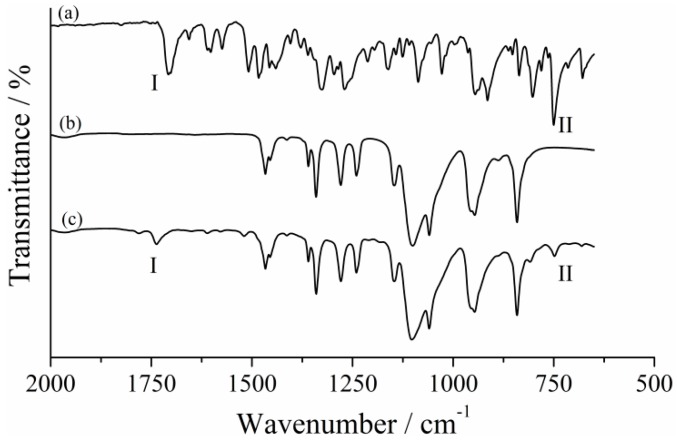
FTIR-ATR spectra of (**a**) SPCOOH, (**b**) PEG 2000 and (**c**) PEGSP2.

The FTIR-ATR spectroscopy is generally used for structural characterization and identification of functional groups in a variety of compounds. In order to investigate the polymer chain modification accomplishment, a comparison between the FTIR-ATR spectra of PEG 2000, its esterification product (PEGSP2) and the SPCOOH is shown Figure 2.

The spectrum of SPCOOH, Figure 2a, shows two main bands region I (from 1750 to 1630 cm^−1^) and region II (from 770 to 685 cm^−1^) that were used for identifying polymer chain modification. The band in the region I of (a) was assigned as carbonyl stretch from the acid moiety of SPCOOH (1708 cm^−1^), and the band in region II was assigned as C_ar_—H stretch at (741 cm^−1^). Two new bands appear after the PEG 2000 (Figure 2b) esterification as expected and depicted in Figure 2c. The first one could be attributed to an ester carbonyl stretch (ν C=O) at 1736 cm^−1^, region I spectrum and the second band showed up in 746 cm^−1^ and corresponds to C_ar_—H stretch, region II. These data demonstrated the SPCOOH chemical modification of PEG 2000 chain, since these bands are not observed in the unmodified polymer spectrum, Figure 2b. The low intensity of these new bands is associated with the polymer molecular weight (Mw). As the polymers Mw increases, the bands associated with the vibrational modes of OH and C—OH decreases in intensity [25]. After the PEG 2000 esterification, it is possible to note a short decrease of O—H stretching intensity (See Supplementary Material—Figure S1).

NMR was carried out to confirm the chemical modification of the PEG 2000 chain by the SPCOOH molecule. The ^1^H-NMR spectra of PEG 2000 and PEGSP2 are presented in Figures S2 and S3, respectively. The PEG 2000 chemical shift (δ) for the CH_2_ hydrogens are in accordance with the literature (δ 3.59) [26]. These hydrogens were observed at δ 3.62 for PEGSP2 after PEG 2000 chemical modification, indicating a slight chemical shift compared to pure polymer. The SPCOOH hydrogens can be observed in the PEGSP2 spectrum Figure S3. Aromatic hydrogens δ from 5.5 to 8.0 could also be integrated confirming the presence of the photochromic dye in the polymer chain as depicted in Figure S3.

Electronic spectroscopy was also used to evaluate the post-polymerization modification of PEG 2000. Figure S4 demonstrates that PEG 2000 did not show significant absorption in the wavelengths (200–800 nm) used for analysis; however, it is possible to observe two main bands assigned in region I and II for the PEGSP2. The electronic transitions at region I (chromene moiety) region II (indoline moiety) are described as a π-π* transition between molecular orbitals [27,28]. Density Functional Theory studies reported in the literature claim that most of LUMO orbitals are distributed over the chromene moiety, being those HOMO orbitals mainly in the indoline moiety depending on the substituents bonded to the indolic nitrogen [29,30]. Thus, UV-vis analysis was crucial to confirm the number of PEG 2000 hydroxyl groups substitution in the polymer structure and to elucidate the possible binding sites between PEGSP2 and metal ions. Based on the results pointed out in Figure S5, the concentration of SPCOOH was determined to be 0.064 mmol L^−1^ in the solution containing PEGSP2 at 0.032 mmol L^−1^, this result suggests esterification in both the hydroxyls of PEG 2000 molecules. A calibration curve was made using SPCOOH as standard, then, the spectrum of PEGSP2 (0.032 mmol L^−1^) was recorded to determine a SPCOOH concentration per molecule of PEGSP2.

### 2.2. Polymers (PEG 2000 and PEGSP2) Thermal Analysis Characterization

Thermal analysis is a useful tool for studying thermal events such as melting temperature (T_m_), glass transition temperature (T_g_) and crystallization temperature (T_c_). The differential scanning calorimetry (DSC) curves show a single melting peak for PEG 2000 or PEGSP2, Figure S6. Both peaks indicate an endothermic fusion for the polymers (PEGSP2 and PEG 2000). The T_m_ obtained for the polymers were 39 °C for PEGSP2 and 50 °C for PEG 2000. The T_m_ for PEG 2000 is consistent with data previously reported in the literature [31]. Latent heat (Δ_fus_H) was calculated for the polymers (see Tables S1 and S2 in Supplementary Material 7) were 9.376 J g^−1^ and 3.424 J g^−1^ for PEG 2000 and PEGSP2, respectively. These results also indicate the substitution of the hydroxyls of the polymer chain, since the bulky molecules of SP shall affect the intermolecular interactions between polymeric chains. Additionally, by analyzing the first derivate of the thermograms in the thermogravimetric analysis (TGA) with respect to time, it is possible to obtain the temperature at which degradation is faster (T_peak_), the temperature where the event starts (T_onset_) and the temperature in which the event ends (T_offset_). For PEGSP2 (Figure S8a), the first thermal event that initiates the degradation is assigned at 228 °C (T_peak_) and for PEG 2000 the T_peak_ of degradation starts at 301 °C (Figure S8b), similar thermograms were observed in the literature [32]. A comparison between the TGA of PEG 2000 and PEGSP2 shows a displacement of T_onset_ and T_offset._ This displacement is expected due to the higher population of carbon/oxygen and carbon/nitrogen bonds present in the SPCOOH moiety; thus, increasing the population of weak bonds causes, an earlier onset of degradation for the functionalized polymer (PEGSP2).

### 2.3. Electrospun Nanofibers Characterization

The distribution of the polymers in the ES nanofibers were characterized by FTIR-ATR spectroscopy. A comparison between FTIR-ATR spectra of pure PCL nanofibers, PCL + PEGSP2 30 % wt nanofibers and PEGSP2 polymer is presented in Figure S9. Bands of PEGSP2 polymer could not be detected in the FTIR-ATR spectrum of PCL + PEGSP2 30 % wt nanofibers. The spectrum of pure PCL ES fibers did not demonstrate significant differences when compared to the polymeric blend. These data can be correlated due to the amount of PCL present in the blend material.

**Figure 3:**
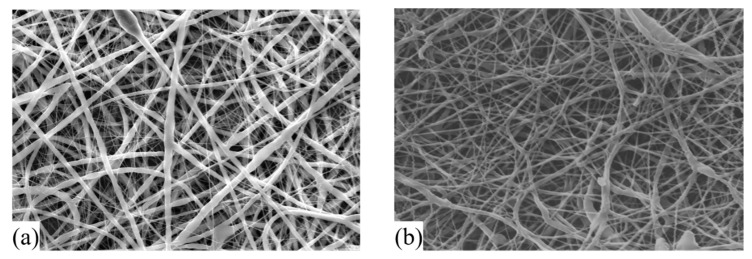
SEM images for the (**a**) poly(ε-caprolactone) (PCL) and (**b**) PCL + PEGSP2 30 % wt nanofibers.

In order to characterize the fibers’ diameters and morphology, SEM was carried out and the results are presented in Figure 3a,b, for PCL and PCL + PEGSP2 30 % wt fibers, respectively. The average diameter calculated for ES nanofibers of PCL was 284 ± 89 nm, and a higher size distribution was also found to be 1.82 ± 0.18 µm. The blend (PCL + PEGSP2 30 % wt fibers) size distribution was determined 246 ± 69 nm, with a narrow distribution. By observing the SEM images, Figure 3, it is possible to see a smooth surface for both fibers. Furthermore, no beads were observed, and in both cases, fibers present a well-defined cylindrical shape with no pores or fractures.

**Figure 4:**
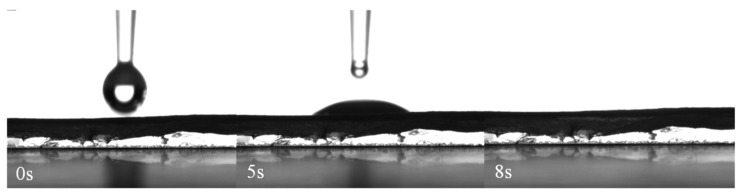
Frames from drop shape analysis of water on the ES nanofibers surface for PCL+ PEGSP2 30% wt.

The surface properties of the ES nanofibers were also characterized by water contact angle (CA) measurements. In the DSA analysis water was used because the CA of water with the material is related to the roughness and the chemical components on the surface. Figure 4 depicts selected frames of a video made using DSA software. It takes approximately eight seconds for the water droplet to be completely absorbed by the ES nanofibers surface.

This wettability is associated with the presence of PEGSP2 in the polymer matrix. However, comparing this result with that obtained for pure PCL nanofibers (Figure S10), CA measurements of water on the surface of pure PCL nanofibers was 131 ± 10 °C; similar CA results were described in the literature [33,34]. Despite the poor solubility of PEGSP2 in water, its presence in the polymeric blend with PCL confers high wettability to the surface. These characteristics could be associated with the increase in population of more polar functional groups present in PEGSP2. These findings are important, because higher interaction between metal ions from solution and the fibers surface is desired in order to increase chemical sensing.

### 2.4. Photochromic Properties and Chemical Sensing of the ES Nanofibers

**Figure 5:**
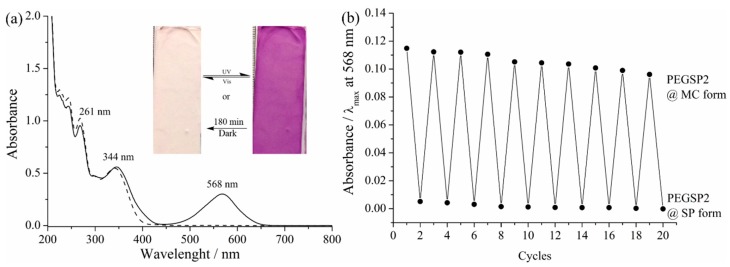
(**a**) Electronic spectra of PEGSP2 isomers (dash line) for closed form - SP, and (solid line) for open form - MC in MeCN. Insert photograph of ES nanofibers after visible light irradiation and after UV irradiation and (**b**) absorbance measurements of irradiation cycles (visible and UV lights) in PEGSP2 MeCN solution.

Photochromic behavior of PEGSP2 is depicted in Figure 5 in solution and solid state when associated with PCL (ES nanofibers). The electronic spectra in MeCN of both isomers were obtained by emitting UV or visible light sources, the band at 568 nm being the major difference, for the open form isomer. The insert at Figure 5a shows that the dye in the ES nanofibers can be isomerized by UV (purple material) and visible light (white material) sources. Sunlight can also induce isomerization (see video Supplementary Material). The isomerization process from open form to closed form takes 180 min if the material is not subjected to any external stimuli, such as light irradiation or heat. In addition, to these results, the PEGSP2 solution was irradiated by cycling UV and visible lights, Figure 5b, demonstrating that PEGSP2 could have a better isomerization efficacy than SPCOOH, after 10 cycles there is a 16.2% decrease in absorbance. Recent literature [35,36,37] also reports SP dyes as sensors, mainly in solution. Based on our data, twenty-five metals ions in aqueous solution were tested on the PEGSP2 nanofibers surface.

**Figure 6:**
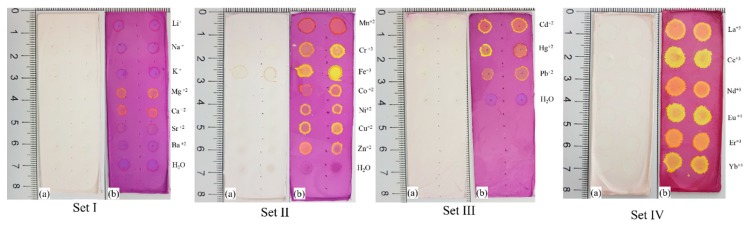
Photographs the photochromic nanofibers after 2 μL metals aqueous solution spots for set I, II, III, and IV. Metal ions spots after (**a**) visible light irradiation and (**b**) after UV light irradiation.

Several experiments were performed with metal ions in solution on the ES nanofibers surface, Figure 6, Set I to Set IV (a) and (b). In order to investigate the counterions’ effect on the nanofibers surface, metals were used based on the Cl^−^ and NO_3_^−^ counterions; however, no significant color change was observed for those metals with color response. The chemical sensing observed for these data could be attributed to the metal ion interaction with the polymer matrix. For Set I, no metals were detectable under visible light conditions; however, two-color response could be observed when Ca^2+^ and Mg^2+^ ions were spotted on the nanofibers surface and the matrix was exposed to the UV light irradiation. These two ions are important to monitor since they are responsible for hard water occurrence. For Set II (a), the Fe^3+^ ion appears as a brown spot when ES nanofibers were irradiated with visible light. In the same Set II (b), under UV light, it is possible to observe two main colors: yellow or orange. Similar color response was observed for Set III and Set IV. Another characteristic observed was the higher wettability of the surface when the lanthanides metal ions solutions were spotted compared to those ions from Set I to Set III.

**Figure 7:**
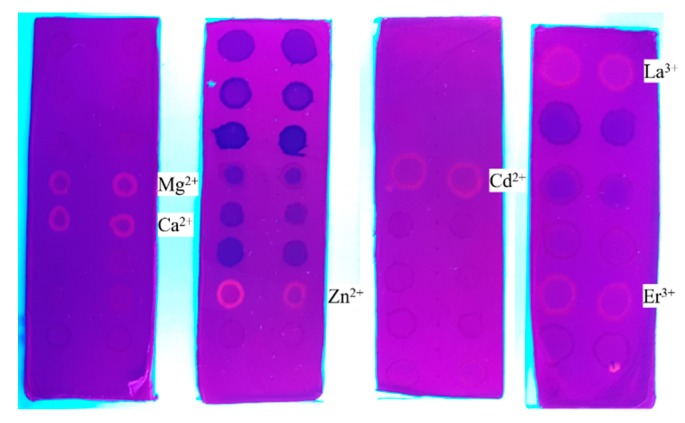
Photograph of ES nanofibers under UV light conditions.

As an important additional result, some of the metal ions tested presented a fluorescence under UV light irradiation (Figure 7), including Ca^2+^ and Mg^2+^. Other researchers have also reported this phenomenon for these ions [35,38]. Zn^2+^ appears orange in color, and it was the only one in its set that presented fluorescence. Zn^2+^ ions have been coordinated to SP molecules and their fluorescence has been described [39]. In Set III, Cd^2+^ was the only one with fluorescent properties; similar spiropyran derivates also shows fluorescence for Cd^2+^ [40]. For Set IV, La^3+^ and Er^3+^ were the metal ions which demonstrated fluorescence under UV irradiation [41]. As can be observed in Figure 7, the other metals did not have fluorescent properties.

As soon as color change was observed for the ES nanofibers with the metal ions, limit detection was determined in a PEGSP2 solution containing those metal ions. Thus, successive injections of metal salts solutions (at 10.00 mmol L^−1^) in a PEGSP2 (at 0.015 mmol L^−1^) were carried out. These additions of metal ions solution into the PEGSP2 induced positive (hyperchromic effect) or negative (hypochromic effect). Table 1 summarizes the minimal concentration that allows the spectroscopy detection of metal ions in solution. Based on these data, Fe^3+^ is the most sensitive metal ion among all tested followed by Fe^2+^ and Cu^2+^. On the other hand, Er^3+^ and Eu^3+^ are the least sensitive ones in solution.

## 3. Materials and Methods

2,3,3-Trimethylindolenine (98%), 3-Iodopropanoic acid (95%), 2-Hidroxy-5-nitrobenzaldehyde (98%), 4-Methylpiperidine (96%), Anhydrous tetrahydrofuran (THF) (≥99.9%), Anhydrous diethyl ether (Et_2_O) (≥99.0%), Anhydrous acetonitrile (MeCN) (99.8%), anhydrous N,N-Dimethylformamide (DMF) (>99.8%), Iron (II) chloride tetrahydrate (FeCl_2_·4H_2_O) (>99.5), La(NO_3_)_3_ was kindly donated from IF Sul de Minas and the other lanthanides were obtained by reacting acid with the oxides: Ce_2_O_3_, Nd_2_O_3_, Eu_2_O_3_, Er_2_O_3_ and Yb_2_O_3_, all from Sigma-Aldrich (Cotia, Brazil). Dimethyl sulfoxide (DMSO) (99.5%), Calcium (II) Nitrate tetrahydrate (Ca(NO_3_)_2_·4H_2_O) (>95.5%) from Nuclear Magnesium (II) nitrate hexahydrate (Mg(NO_3_)_2_·6H_2_O) (>95.5%) from Isofar (Duque de Caxias, Brazil), Strontium (II) nitrate (Sr(NO_3_)_2_) (>95.5%), Cobalt (II) nitrate hexahydrate (Co(NO_3_)_2_·6H_2_O) (98%), Cadmium (II) nitrate tetrahydrate (Cd(NO_3_)_2_·4H_2_O) (>95.5%), Lead (II) nitrate (PbNO_3_)_2_ (>95.5%) from Dinâmica (Indaiatuba, Brazil), Barium (II) nitrate (Ba(NO_3_)_2_) (>95.5%), Nickel (II) chloride (NiCl_2_·6H_2_O) (>95.5%), Copper (II) nitrate hexahydrate (Cu(NO_3_)_2_·6H_2_O) (>95.5%), Copper (II) chloride hexahydrate (CuCl_2_·6H_2_O) (>95.5%), Zinc (II) nitrate hexahydrate (Zn(NO_3_)_2_·6H_2_O) (>95.5%), Zinc (II) chloride anhydrous (ZnCl) (>95.5%) from Neon (Suzano, Brazil), Chromium (III) nitrate nonahydrate (Cr(NO_3_)_3_·9H_2_O) (>95.5%), Chromium (III) chloride hexahydrate (CrCl_3_·6H_2_O) (>95.5%), Manganese (II) nitrate hexahydrate (Mn(NO_3_)_2_·6H_2_O) (>95.5%), Manganese (II) chloride hexahydrate (MnCl_2_·6H_2_O) (>95.5%), Ferric nitrate nonahydrate (Fe(NO_3_)_3_·9H_2_O) (>95.5%) Iron (III) chloride hexahydrate (FeCl_3_·6H_2_O) (>95.5%), Cobalt (II) chloride dihydrate (CoCl_2_·2H_2_O) (>95.5%), Mercury (II) nitrate monohydrate (Hg(NO_3_)_2_·6H_2_O), Nickel (II) nitrate hexahydrate (Ni(NO_3_)_2_·6H_2_O) (>95.5%) from Vetec (Duque de Caxias, Brazil), poly(ε-caprolactone) (PCL) of 43.000–50.000 g mol^−1^ and poly(ethylene glycol) 2.000 g mol^−1^ were purchased from Polysciences (Warrington, PA, USA), all used without further purification.

### 3.1. Polymer Modification

Carboxylic acid spiropyran (SPCOOH) or (l-(β-Carboxyethyl)-3′,3′-dimethyl-6-nitrospiro(indoline-2′,2[2*H*-1] benzopyran), was synthesized using a similar methodology to the one described previously [42]. PEGSP2 was prepared by dissolving 2 equivalents of SPCOOH in anhydrous THF under N_2_ atmosphere with dicyclohexylcarbodiimide (DCC) to form the intermediate and *N*-hydroxysuccinimide (NHS) as a catalyst. The solution was kept under stirring at 45 °C. After 45 min, PEG 2000 previously dissolved in anhydrous THF, was added to the reaction. The initial color was blue and after the addition, it turned to purple. After 4 h a purple powder was obtained from precipitation in anhydrous Et_2_O. Then, the product was washed with EtOH and Et_2_O and solid was kept in a desiccator for 24 h to completely dry.

### 3.2. Electrospun Fibers

Photochromic nanofibers were prepared using a 30% *w*/*v* of PCL, in a solution containing 60:40 of THF/DMF. PEGSP2 was added in a ratio of 30% *w*/*w*. The solutions were kept under constant stirring for 2 days to ensure the complete polymer dissolution. An electrospinning apparatus consisting of a 12 mL syringe connected to a syringe pump (Harvard Apparatus PHD 2000 infusion, Holliston, MA, USA) was used to supply a steady flow of 1.8 mL h^−1^ of solution to the tip of the needle. A high-voltage power supply (Gamma High Voltage, Ormond Beach, FL, USA) was used to apply the potential of 19 kV to the syringe needle. The target consisted of a grounded steel plate of 20 × 20 cm placed 18 cm from the needle tip to ensure that fibers were dry upon collection [17].

### 3.3. Experimental Characterization

All FTIR-ATR spectra were recorded using a Perkin Elmer Spectrum 100 instrument (São Paulo, Brazil) equipped with a diamond/ZnSe crystal with attenuated total reflectance (ATR) module (wavelength range from 4000 to 650 cm^−1^, 4 cm^−1^ of resolution and 64 scans).

UV-visible (UV-Vis) absorption spectra were obtained at 20 ± 1 °C. Spectra were measured in the UV-Vis range (from 200 to 800 nm) using a Varian Cary 50 Scan spectrophotometer (Santa Clara, CA, USA) with quartz cuvettes with a path length of 10 mm and a volume of 1.0 mL. Spectra were recorded using MeCN, DMSO and DMF, to characterize PEGSP2. The scan rate used for all analysis was 2400 nm min^−1^. The reversibility experiment for PEGSP2 was carried out in MeCN and the quartz cuvette was sealed to avoid solvent evaporation. Ten cycles were performed and each cycle consisted of 30 s of irradiation with a UV source (λ = 365 nm) followed by 30 s of irradiation with a LED light (λ = 436 nm). A titration between of each metal ion in the SPCOOH solution was carried out. The successive injections of metal solutions were all performed in MeCN, except for Fe^2+^ which was made in DMF and for Mg^2+^, Ca^2+^, Ce^3+^ and Yb^3+^ (carried out in DMSO). For all titrations, the concentration of PEGSP2 in the cuvette was 0.015 mmol L^−1^ and the volume of metal ion solution was 2.0 µL.

Nuclear magnetic resonance spectroscopy of ^1^H was performed in a Bruker 600 MHz (Billerica, MA, USA) using tetramethylsilane as internal standard and chloroform (CDCl_3_) was used as solvent.

Thermogravimetric analysis was performed in a METTLER TG 50 (Columbus, OH, USA) with a METTLER MT5 balance and a METTLER TC 11 TA PROCESSOR controller under N_2_ atmosphere at 20 mL min^−1^ from 25 to 1000 °C. The heating rate for TGA was 10 °C min^−1^. Differential scanning calorimetry experiments were carried out in a DSC-60 Shimadzu with a closed aluminum melting pot under N_2_ atmosphere with a flow rate of 2 mL min^−1^ from 25 to 150 °C. The heating rate was 1 °C min^−1^.

Surface morphology of the ES fibers was characterized by SEM operated at 14–15 kV. Dried samples were gold-coated for 2 min, with 6 mA current using an IC-50 ion coater Shimadzu. The fibers diameter was determined using at least 30 measurements from different micrographs.

DSA was done in a Krüs GmbH Germany (Hamburg), model FM40MK2 Easy Drop using water as solvent. CA measurements were carried out 5 times, using 10 μL of water.

The spots in the colorimetric experiment were made using a micropipette using 2 μL of metal solution in all cases. Samples were categorized from Set I to Set IV based on the ions characteristics (Set I: alkaline and alkaline earth metals, Set II: transition metals: Set III: heavy metals and Set IV: rare earth metals) ES nanofibers were obtained on the microscope glass slides, being irradiated with the UV lamp for 2 min, with a distance of 10 cm. The radiation sources used for the photographs and experiments were UV lamp (λ = 365 nm, 20 W) and visible light LED source (λ = 436 nm, 40 W). Reaction between metal ions and ES nanofibers surface was immediately observed.

## 4. Conclusions

The present work comprised the functionalization and characterization of a PEG by post-polymerization esterification with an acid spiropyran derivate. Electrospun nanofibers consisting of a polymeric blend between PCL and PEGSP2 were characterized and tested as a metal ion sensor. Eighteen metals ions changed the color of the ES nanofibers. Six metal ions demonstrated orange fluorescence under UV light, and most of the transition metals and rare-earth metals showed a yellowish or orange color. The modified PEG presented sensitivity for metal ions in the solid state and in solution. The minimal detectable concentration of metal ions using PEGSP2 in solution (0.015 mmol L^−1^) was observed for Fe^3+^, which can be detected from 0.005 g L^−1^.

## Figures and Tables

**Table 1:** Metal ions and their minimum concentration detected by PEGSP2 in solution. Wavelength of maxima absorption (λmax) chosen was 349 nm.

Metal (M^n+^)	Minimum Concentration for Detection (mmol L^−1^)	Spectral Effect
Mg^2+^	0.692	hypochromic
Ca^2+^	0.163	hyperchromic
Cr^3+^	0.024	hypochromic
Mn^2+^	0.037	hyperchromic
Fe^3+^	0.009	hypochromic
Fe^2+^	0.016	hypochromic
Co^2+^	0.040	hyperchromic
Ni^2+^	0.055	hyperchromic
Cu^2+^	0.025	hypochromic
Zn^2+^	0.037	hypochromic
Cd^2+^	0.156	hypochromic
La^3+^	1.250	hypochromic
Ce^3+^	0.117	hyperchromic
Nd^3+^	1.130	hypochromic
Eu^3+^	2.602	hypochromic
Er^3+^	1.804	hypochromic
Yb^3+^	0.147	hypochromic

## Supplementary Materials

The following are available online, S1: FTIR-ATR spectra of SPCOOH, PEG 2000 and PEGSP2, S2 ^1^H-NMR spectrum of PEG 2000 in CDCl_3_, at 600 MHz and 27 °C, S3 ^1^H-NMR spectrum of PEGSP2 in CDCl_3_, at 600 MHz and 27 °C and proposed structure for PEGSP2, S4 electronic spectra of PEG 2000 at 2.1 mmol L^−1^ and PEGSP2 at 0.062 mmol L^−1^, S5. (**a**) Electronic spectra of SPCOOH diluitons from 0.014 to 0.71 mmol L^−1^ (solid lines) and PEGSP2 electronic spectrum at 0.031 mmol L^−1^ (dot line) and (**b**) calibration curve and linear fit for SPCOOH. All spectra were obtained in MeCN solvent using the maximum wavelength at 341 nm, S6 DSC curves for PEG 2000 and PEGSP2, S7 Table S1 Calculations of Δ_fus_H of PEGSP2, Table S2 Calculations of Δ_fus_H of PEG 2000, S8. (**a**) TGA/DTG of PEGSP2 and (**b**) TGA/DTG of PEG 2000, S9. FTIR-ATR spectra of (**a**) PEGSP2 polymer, and electrospun fibers of (**b**) PCL + PEGSP2 30 % wt and (**c**) pure PCL, S10. DSA (water contact angle) for pure PCL electrospun nanofibers, Video S1: Sunlight irradiation.

## References

[1] Shang J., Lin S., Theato P. (2017). Fabrication of color changeable CO_2_ sensitive nanofibers. Polym. Chem..

[2] Kulkarni S.B., Navale Y.H., Navale S.T., Stadler F.J., Patil V.B. (2019). Room temperature ammonia gas sensing properties of polyaniline nanofibers. J. Mater. Sci. Mater. Electron..

[3] Adhikari B., Majumdar S. (2004). Polymers in sensor applications. Prog. Polym. Sci..

[4] Lakshmi K., Kadirvelu K., Mohan P.S. (2019). Chemically modified electrospun nanofiber for high adsorption and effective photocatalytic decontamination of organophosphorus compounds. J. Chem. Technol. Biotechnol..

[5] Senthamizhan A., Balusamy B., Uyar T. (2016). Glucose sensors based on electrospun nanofibers: A review Fiber-based Platforms for Bioanalytics. Anal. Bioanal. Chem..

[6] Xue R., Behera P., Xu J., Viapiano M.S., Lannutti J.J. (2014). Sensors and Actuators B: Chemical Polydimethylsiloxane core—Polycaprolactone shell nanofibers as biocompatible, real-time oxygen sensors. Sens. Actuators B Chem..

[7] Deniz E., Tomasulo M., Cusido J., Sortino S., Raymo F.M. (2011). Fast and stable photochromic oxazines for fluorescence switching. Langmuir.

[8] Li B.D., Xia Y. (2004). Electrospinning of Nanofibers: Reinventing the Wheel?. Adv. Mater..

[9] Reneker D.H., Chun I. (1996). Nanometre diameter fibres of polymer, produced by electrospinning. Nanotechnology.

[10] Khatri Z., Ali S., Khatri I., Mayakrishnan G., Hun S., Kim I. (2015). UV-responsive polyvinyl alcohol nanofibers prepared by electrospinning. Appl. Surf. Sci..

[11] Yoon J., Chae S.K., Kim J.M. (2007). Colorimetric sensors for volatile organic compounds (VOCs) based on conjugated polymer-embedded electrospun fibers. J. Am. Chem. Soc..

[12] Thornton B.T.E., Harrison A., Pham A.L., Castano C.E., Tang C. (2018). Polyaniline-Functionalized Nanofibers for Colorimetric Detection of HCl Vapor. ACS Omega.

[13] Wang X., Drew C., Lee S.H., Senecal K.J., Kumar J., Samuelson L.A. (2002). Electrospun Nanofibrous Membranes for Highly Sensitive Optical Sensors. Nano Lett..

[14] Ongun M.Z., Ertekin K., Gocmenturk M., Ergun Y., Suslu A. (2012). Copper ion sensing with fluorescent electrospun nanofibers. Spectrochim. Acta Part A Mol. Biomol. Spectrosc..

[15] Syu J.H., Cheng Y.K., Hong W.Y., Wang H.P., Lin Y.C., Meng H.F., Zan H.W., Horng S.F., Chang G.F., Hung C.H. (2013). Electrospun fibers as a solid-state real-time zinc ion sensor with high sensitivity and cell medium compatibility. Adv. Funct. Mater..

[16] Terra I.A.A., Mercante L.A., Andre R.S., Correa D.S. (2017). Fluorescent and colorimetric electrospun nanofibers for heavy-metal sensing. Biosensors.

[17] Xue J., Wu T., Dai Y., Xia Y. (2019). Electrospinning and electrospun nanofibers: Methods, materials, and applications. Chem. Rev..

[18] Bhardwaj N., Kundu S.C. (2010). Electrospinning: A fascinating fi ber fabrication technique. Biotechnol. Adv..

[19] Gauthier M.A., Gibson M.I., Klok H.A. (2009). Synthesis of functional polymers by post-polymerization modification. Angew. Chem. Int. Ed..

[20] Günay K.A., Theato P., Klok H.A. (2013). Standing on the shoulders of hermann staudinger: Post-polymerization modification from past to present. J. Polym. Sci. Part A Polym. Chem..

[21] Klajn R. (2014). Spiropyran-based dynamic materials. Chem. Soc. Rev..

[22] Rini M., Holm A.K., Nibbering E.T.J., Fidder H. (2003). Ultrafast UV-mid-IR investigation of the ring opening reaction of a photochromic spiropyran. J. Am. Chem. Soc..

[23] Heiligman-Rim R., Hirshberg Y., Fischer E. (1962). Photochromism in Spiropyrans. Part I-V. J. Phys. Chem..

[24] Wojtyk J.T.C., Wasey A., Kazmaier P.M., Hoz S., Buncel E. (2000). Thermal reversion mechanism of n-functionalized merocyanines to spiropyrans: A solvatochromic, solvatokinetic, and semiempirical study. J. Phys. Chem. A.

[25] Rubinson K.A., Meuse C.W. (2013). Deep hydration: Poly(ethylene glycol) *M*w 2000–8000 da probed by vibrational spectrometry and small-angle neutron scattering and assignment of Δg° to individual water layers. Polymer.

[26] Dust J.M., Fang Z.H., Harris J.M. (1990). Proton NMR Characterization of Poly(ethylene Glycols) and Derivatives. Macromolecules.

[27] . Miguez F.B., Reis I.F., Dutra L.P., Silva I.M.S., Verano-Braga T., Lopes J.F., De Sousa F.B. (2019). Electronic investigation of light-induced reversible coordination of Co(II)/spiropyran complex. Dyes Pigments.

[28] Tyer N.W., Becker R.S. (1970). Photochromic Spiropyrans. I. Absorption Spectra and Evaluation of the π-Electron Orthogonality of the Constituent Halves. J. Am. Chem. Soc..

[29] Kubinyi M., Varga O., Baranyai P., Kállay M., Mizsei R., Tárkányi G., Vidóczy T. (2011). Metal complexes of the merocyanine form of nitrobenzospyran: Structure, optical spectra, stability. J. Mol. Struct..

[30] Minkin V.I. (2004). Photo-, thermo-, solvato-, and electrochromic spiroheterocyclic compounds. Chem. Rev..

[31] Kou Y., Wang S., Luo J., Sun K., Zhang J., Tan Z., Shi Q. (2019). Thermal analysis and heat capacity study of polyethylene glycol (PEG) phase change materials for thermal energy storage applications. J. Chem. Thermodyn..

[32] Sundararajan S., Samui A.B., Kulkarni P.S. (2017). Versatility of polyethylene glycol (PEG) in designing solid-solid phase change materials (PCMs) for thermal management and their application to innovative technologies. J. Mater. Chem. A.

[33] Simões M.C.R., Cragg S.M., Barbu E., De Sousa F.B. (2019). The potential of electrospun poly(methyl methacrylate)/polycaprolactone core–sheath fibers for drug delivery applications. J. Mater. Sci..

[34] Narayanan G., Shen J., Boy R., Gupta B.S., Tonelli A.E. (2018). Aliphatic polyester nanofibers functionalized with cyclodextrins and cyclodextrin-guest inclusion complexes. Polymers.

[35] Wang L., Yao Y., Wang J., Dong C., Han H. (2019). Selective sensing Ca^2+^ with a spiropyran-based fluorometric probe. Luminescence.

[36] Kumar A., Kumar A., Sahoo P.R., Kumar S. (2019). Colorimetric and Fluorescence-Based Detection of Mercuric Ion Using a Benzothiazolinic Spiropyran. Chemosensors.

[37] Wang Y., Xu Z., Dai X., Li H., Yu S., Meng W. (2019). A New Spiropyran-Based Sensor for Colorimetric and Fluorescent Detection of Divalent Cu^2+^ and Hg^2+^ Ions and Trivalent Ce^3+^, Cr^3+^ and Al^3+^ Ions. J. Fluoresc..

[38] Filley J., Ibrahim M.A., Nimlos M.R., Watt A.S., Blake D.M. (1998). Magnesium and calcium chelation by a bis-spiropyran. J. Photochem. Photobiol. A Chem..

[39] Shao N., Wang H., Gao X., Yang R., Chan W. (2010). Spiropyran-based fluorescent anion probe and its application for urinary pyrophosphate detection. Anal. Chem..

[40] Natali M., Soldi L., Giordani S. (2010). A photoswitchable Zn (II) selective spiropyran-based sensor. Tetrahedron.

[41] Abdel-Mottaleb M.S.A., Saif M., Attia M.S., Abo-Aly M.M., Mobarez S.N. (2018). Lanthanide complexes of spiropyran photoswitch and sensor: Spectroscopic investigations and computational modelling. Photochem. Photobiol. Sci..

[42] De Sousa F.B., Guerreiro J.D.T., Ma M., Anderson D.G., Drum C.L., Sinisterra R.D., Langer R. (2010). Photo-response behavior of electrospun nanofibers based on spiropyran-cyclodextrin modified polymer. J. Mater. Chem..

