# Supplementary material for: Nanostructured and Photochromic Material for Environmental Detection of Metal Ions
Source: Molecules. 2019 Nov 21;24(23):4243. doi: 10.3390/molecules24234243 (PMC6930475; doi:10.3390/molecules24234243)
Supplement: Supplementary file 1 [file molecules-24-04243-s001.zip › Supplementary_Material_MOLECULES_20_11_19.pdf]

Nanostructured and photochromic material for environmental detection of metal ions

Raphael C. L. Machado [1], Frank Alexis [2], Frederico B. De Sousa [1,*]

[1]Laboratório de Sistemas Poliméricos e Supramoleculares (LSPS) −Instituto de Física e Química, Universidade Federal de Itajubá (UNIFEI), Itajubá, 37500-903, MG, Brazil.

[2]School of Biological Sciences and Engineering, Yachay Tech, San Miguel de Urcuquí, Ibarra EC 100150, Ecuador; falexis@yachaytech.edu.ec

*Corresponding author:
Frederico Barros de Sousa
Instituto de Física e Química, Universidade Federal de Itajubá, UNIFEI, Itajubá, 37500-903, Brazil
Phone: 55-35-3629-1757
Email: fredbsousa@unifei.edu.br & fredbsousa@gmail.com

Supplementary Material 1:

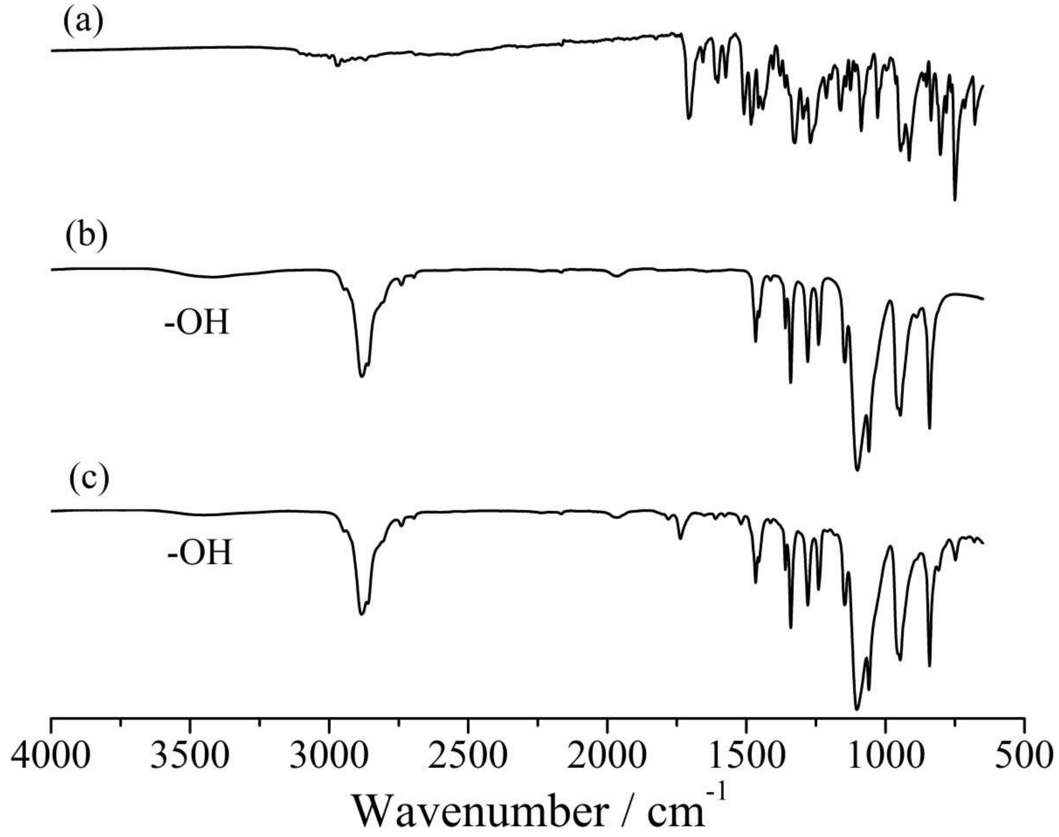

Figure S1. FTIR-ATR spectra of (a) SPCOOH, (b) PEG 2000 and (c) PEGSP2.

Supplementary Material 2:

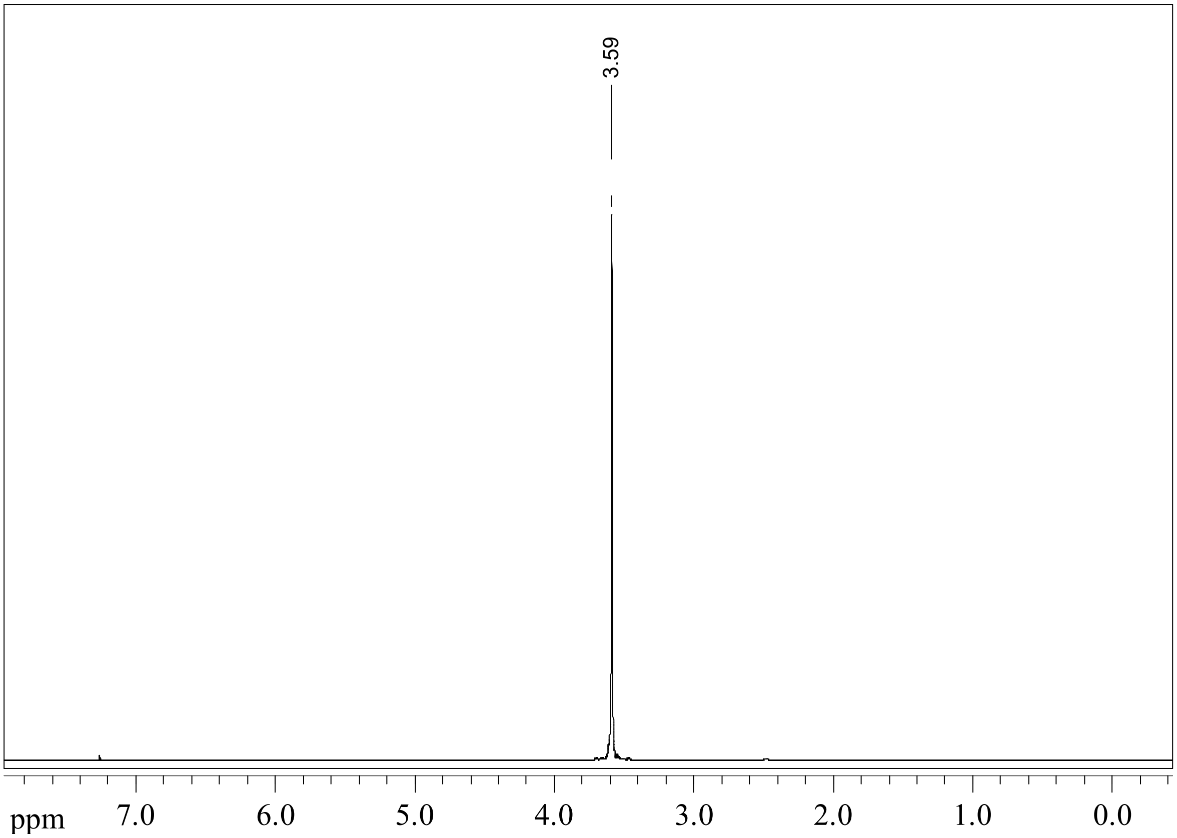

Figure S2. ^{1}H NMR spectrum of PEG 2000 in $CDCl_3$, at 600 MHz and 27 ^{o}C.

Supplementary Material 3:

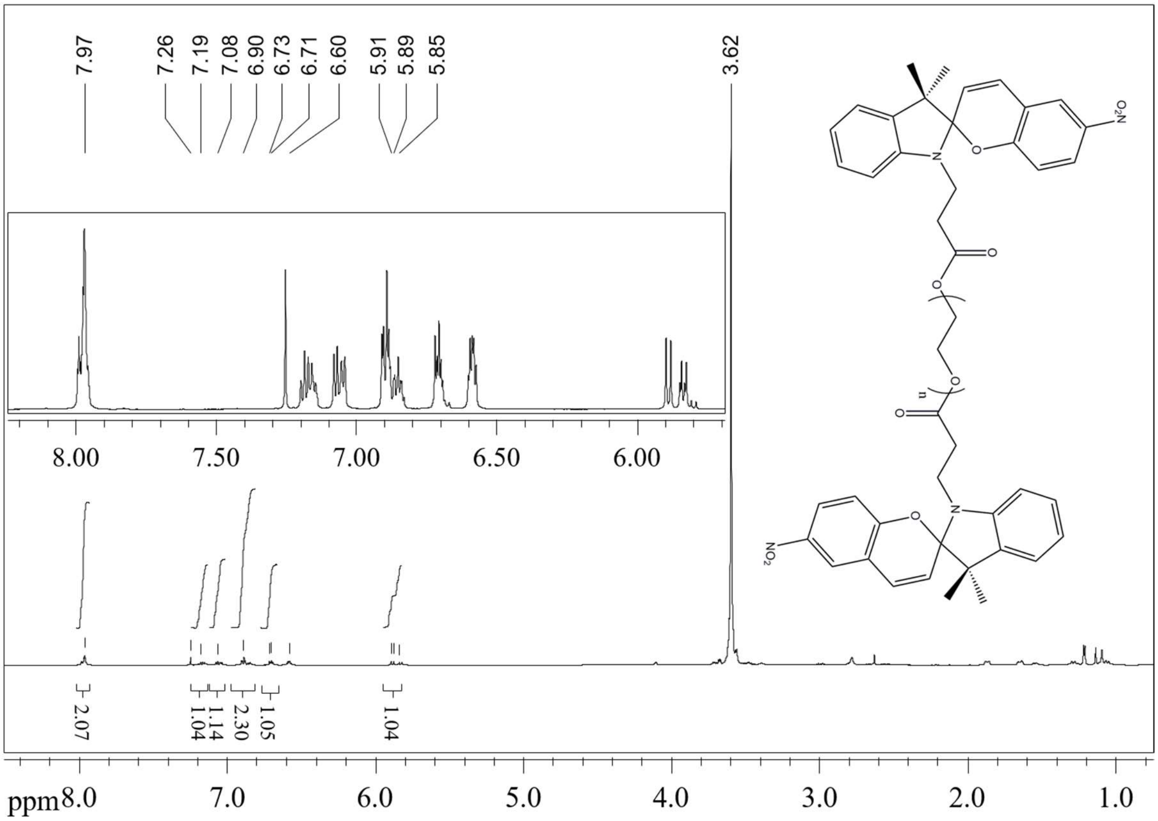

Figure S3. ^{1}H NMR spectrum of PEGSP2 in $CDCl_3$, at 600 MHz and 27 ºC and proposed structure for PEGSP2.

Supplementary Material 4:

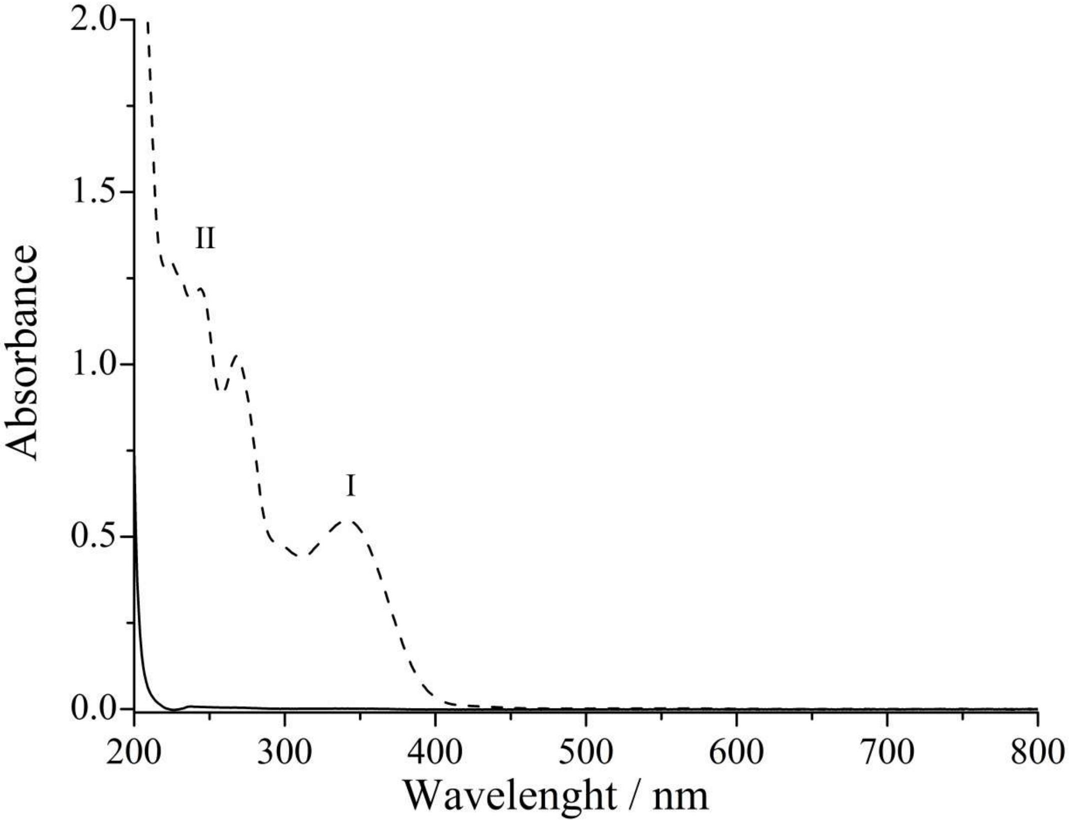

Figure S4. Electronic spectra of PEG 2000 at 2.1 $mmol.L^{-1}$ and PEGSP2 at 0.062 $mmol.L^{-1}$.

Supplementary Material 5:

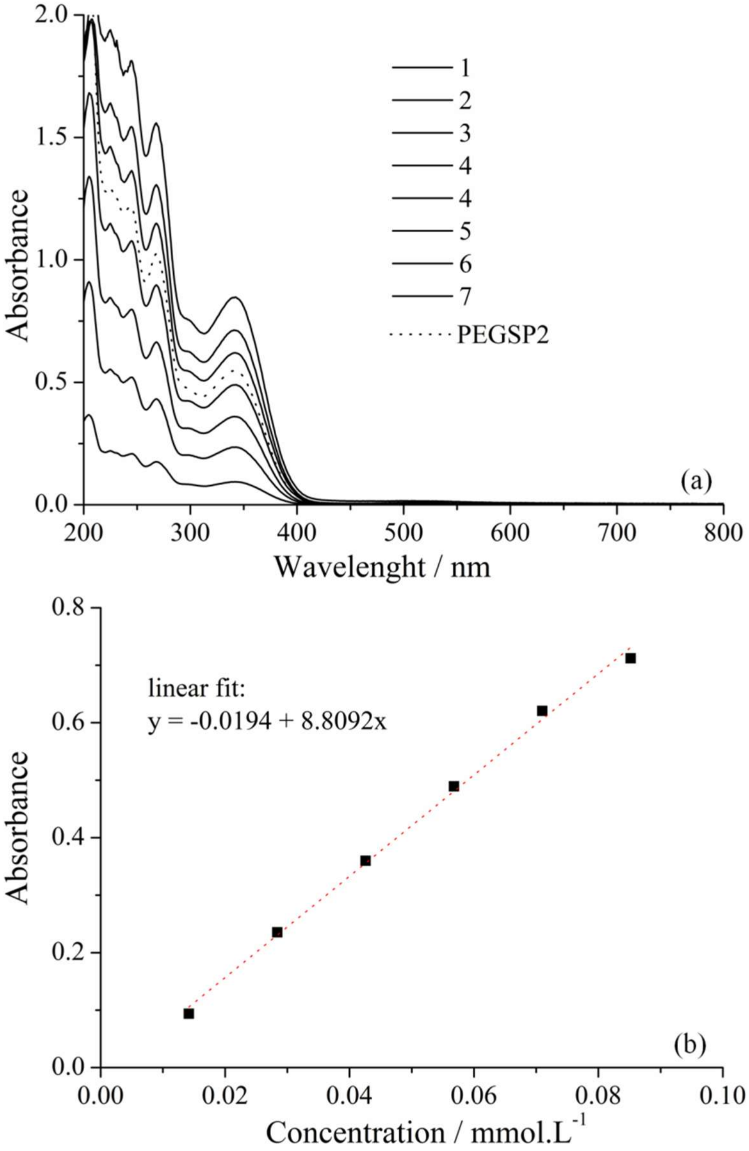

Figure S5. (a) Electronic spectra of SPCOOH diluitons from 0.014 to 0.71 mmol.L^{-1} (solid lines) and PEGSP2 electronic spectrum at 0.031 mmol.L^{-1} (dot line) and (b) calibration curve and linear fit for SPCOOH. All spectra were obtained in MeCN solvent using the maximum wavelength at 341 nm.

Supplementary Material 6:

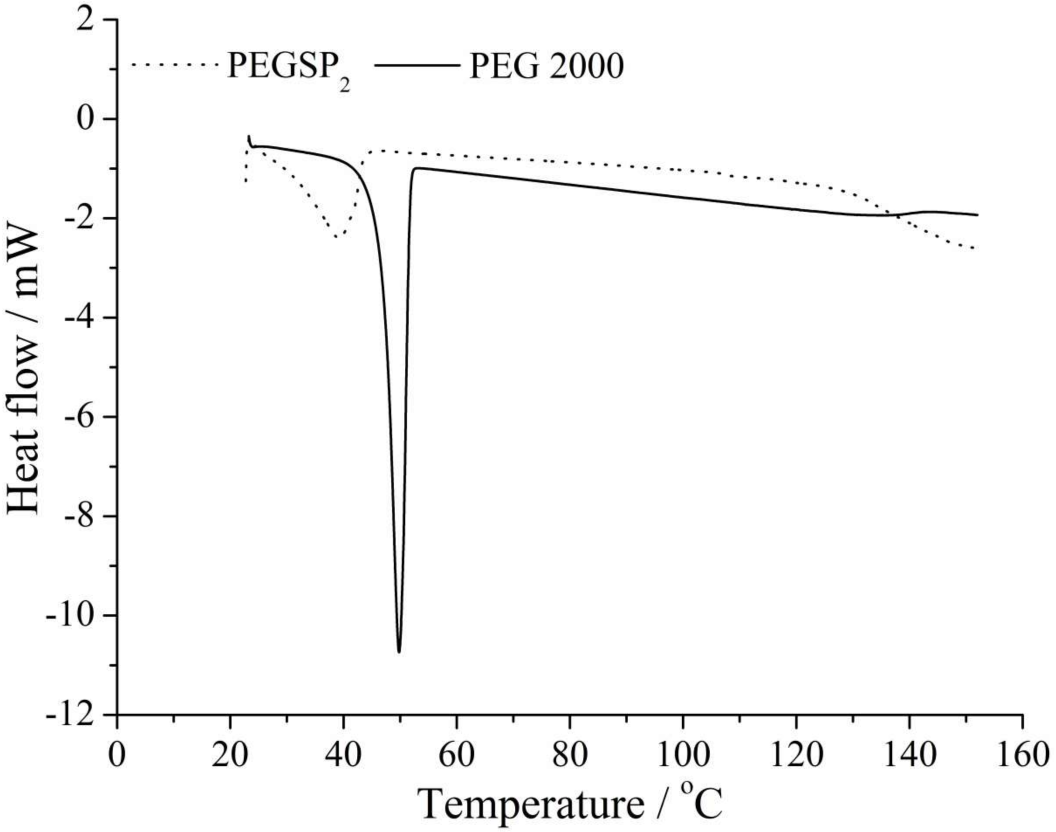

Figure S6. DSC curves for PEG 2000 and PEGSP2.

Supplementary Material 7:

Table S1. Calculations of $\Delta_{fus}H$ of PEGSP2:

Parameters	Values
**Mass**	0.012887 g
**X_1**	23.521673 °C
**X_2**	46.082676 °C
**I_1**	1 s
**I_2**	1463 s
**Area**	–30.183748048742 mW
**X_0**	39.291683 °C
**$\Delta_{fus}H = [Area*(I_1-I_2)]/Mass$**	3.424 $J.g^{-1}$

Table S2. Calculations of DfusH of PEG 2000:

Parameters	Values
**Mass**	0.009951 g
**X_1**	27.507933 °C
**X_2**	53.729233 °C
**I_1**	1 s
**I_2**	1720 s
**Area**	–54.278186629807 mW
**X_0**	49.816132 °C
**$\Delta_{fus}H = [Area*(I_1-I_2)]/Mass$**	9.376 $J.g^{-1}$

Supplementary Material 8:

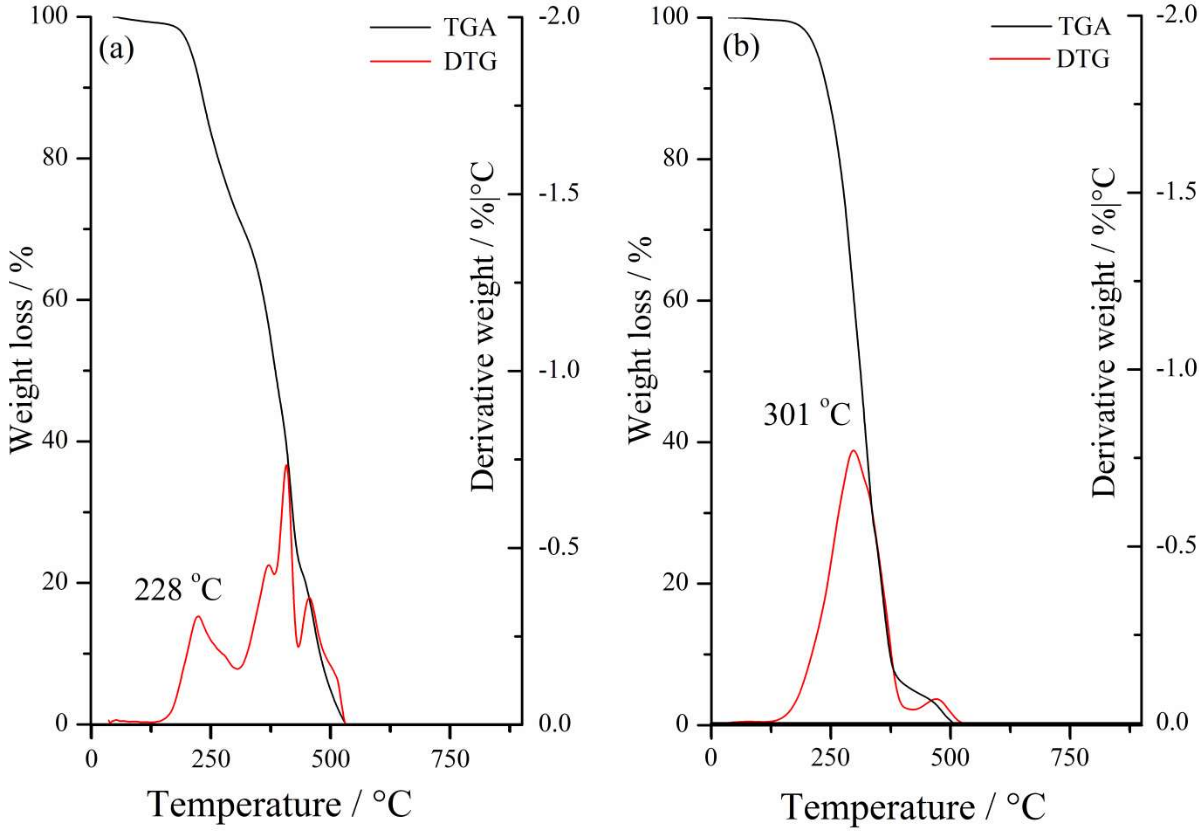

Figure S8. (a) TGA/DTG of PEGSP2 and (b) TGA/DTG of PEG 2000.

Supplementary Material 9:

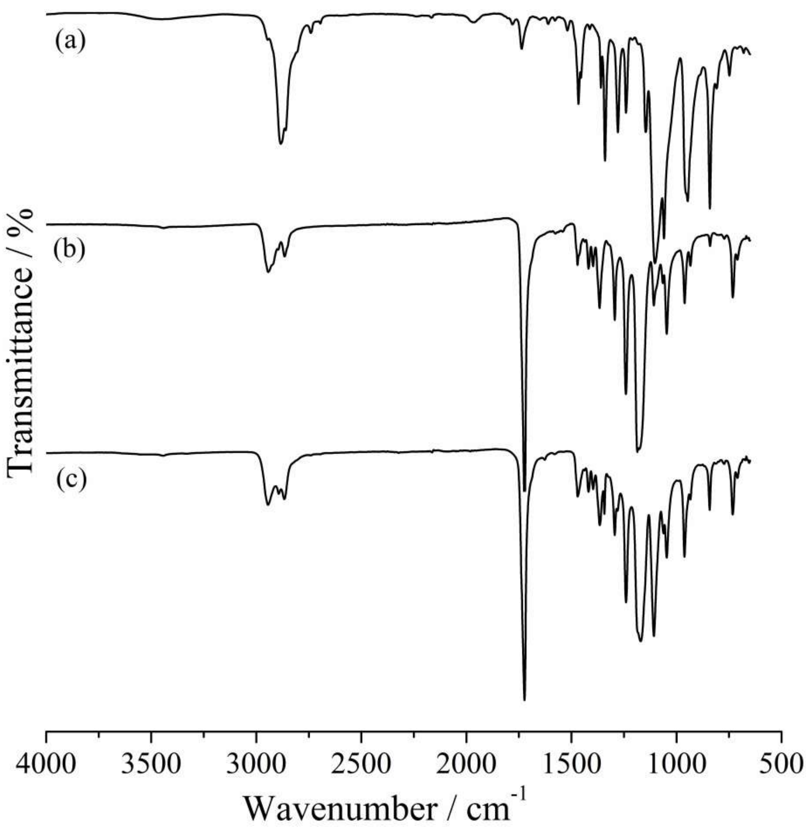

Figure S9. FTIR-ATR spectra of (a) PEGSP2 polymer, and electrospun fibers of (b) PCL + PEGSP2 30% wt and (c) pure PCL.

Supporting Information 10:

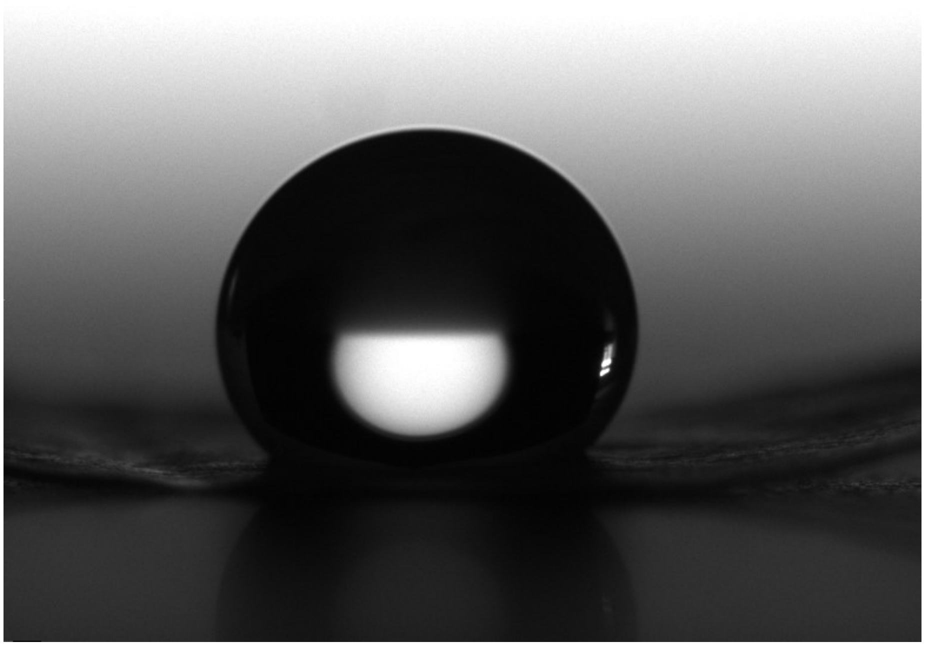

Figure S10. DSA (water contact angle) for pure PCL electrospun nanofibers.
